# Dual roles of anterior cingulate cortex neurons in pain and pleasure in adult mice

**DOI:** 10.1186/s13041-018-0416-1

**Published:** 2018-12-04

**Authors:** Jing-Shan Lu, Qi-Yu Chen, Sibo Zhou, Kaoru Inokuchi, Min Zhuo

**Affiliations:** 1 Center for Neuron and Disease, Frontier Institute of Science and Technology, Xi’an Jiaotong University, Xi’an, 710049 China; 2 Department of Biochemistry, Graduate School of Medicine and Pharmaceutical Sciences, University of Toyama, 2630 Sugitani, Toyama, 930-0194 Japan; 3 Departmentof Physiology, Faculty of Medicine, University of Toronto, 1 King’s College Circle, Toronto, Ontario M5S 1A8 Canada

**Keywords:** IEG, ACC, *Arc*, *Homer1a*, Pain, Sexual attraction

## Abstract

Human and animal studies indicate that some brain regions are activated during painful and pleasant situations, such as the anterior cingulate cortex (ACC). In the present study, we wanted to determine if some of the same neurons in the ACC may be activated by both pain and pleasure. We labeled neurons activated by two stimuli by using two immediate early genes (IEGs), *Arc* and *Homer1a*, and detected the intranuclear transcription of the IEG mRNA in situ. We found that there are double-labeling neurons in the ACC after the mice received pain and sexual attraction stimulation. The double-labeling ACC neurons were higher in male mice exposed to female mice (attractive stimulus) than the group exposed to male mice (normal stimulus). The IEG, which indicates the sexual attraction, were also higher in the female exposing group, while the IEG indicating pain showed no significant variance between two groups. Our findings suggest that ACC neurons play important roles in the process of both pain and pleasure.

## Main text

Pain and pleasure are two major experiences in human and animals. We often pursue pleasure, while avoiding pain. The relationship between pain and pleasure can be further demonstrated by the analgesic effect of pleasure. Palatable food, pleasant odor, pleasurable music and sexual behavior were all found to reduce pain [1]. More interestingly, in certain conditions, pleasure can be gained by enduring pain, such as enjoying a spicy hotpot or receiving a massage. The central mechanism for pleasure is less investigated than the mechanism for pain, which has received more attention [2, 3]. The ACC is well known to participate in pain sensation and nociceptive processing [4–7]. Less has been studied about the ACC’s relationship to pleasure or happiness. It has been reported that the ACC is activated when a man views the picture of his lover [8]. Wu et al. has reported that ACC neurons of male mice could be activated after exposed to female mice [9]. However, it is unclear if pain or pleasure are processed through different population of neurons, or if some ACC neurons may contribute to both pain and pleasure.

CatFISH serves as a functional imaging that allows investigators to distinguish neuronal populations activated by two distinct stimuli. The IEGs such as *Arc*, *Homer1a*, *zif268* and *c-fos*, were used in catFISH. Among them, the *Arc*/*Homer1a* catfish method is a useful tool [10–12]. In this study, we used the *Arc*/*Homer1a* catFISH to distinguish two (pain and sexual attraction) stimuli and to identify ACC neurons that are activated by both stimuli.

**Fig. 1:**
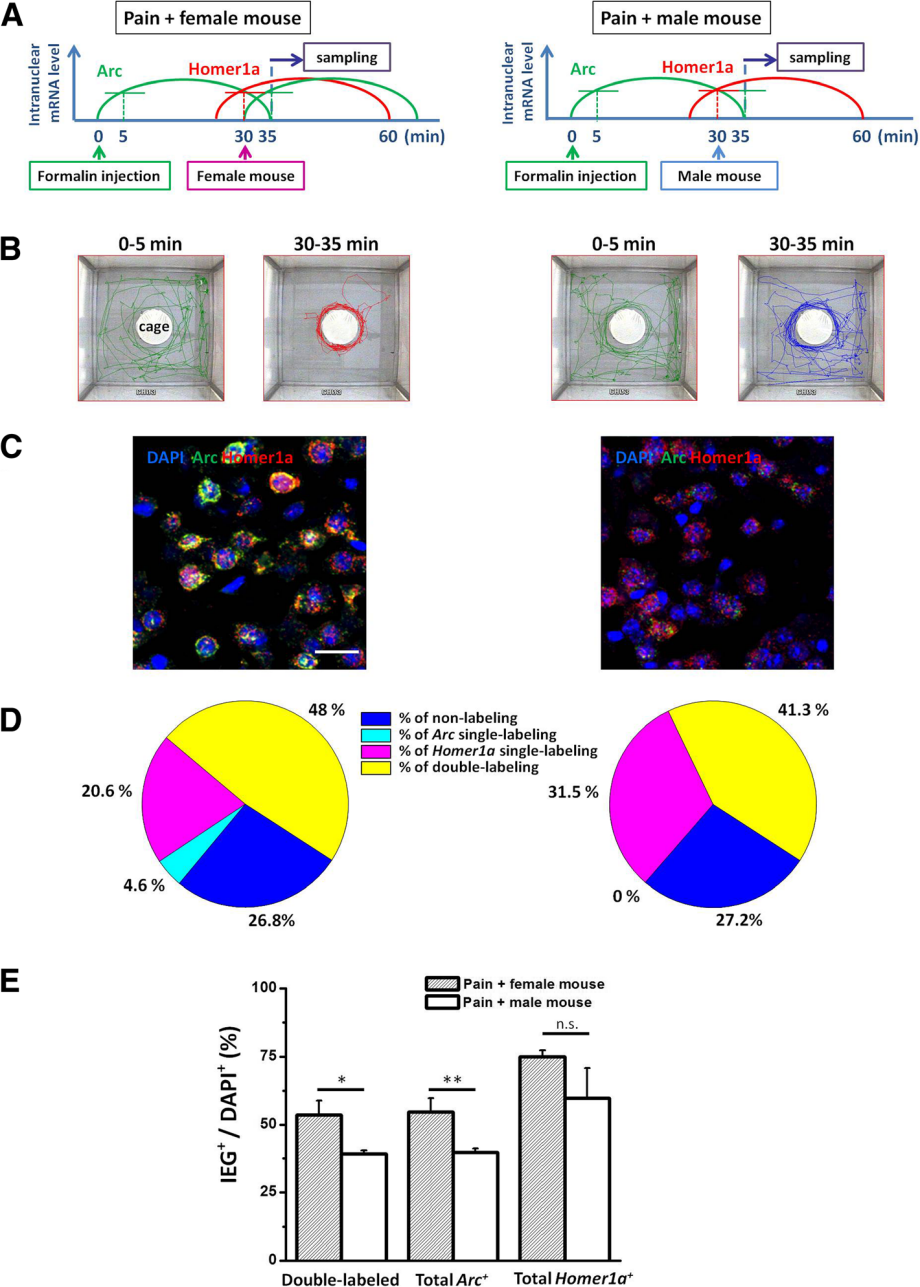
Neurons encoding pain and sexual attraction information were co-labeled in the ACC of mice. **a** Stimulation patterns to induce the expression of the IEGs *Arc* and *Homer1a*. 5% formalin for 5 μl was injected into the left hind paw of the mice. Then the behaviors of the mice in the open field with a cage in the center were recorded. A female (Left) or male (Right) mouse was put into the central cage for 30 min, and the mice were sacrificed at 35 min. **b** The tracks of the “pain + female mouse” group (left) and the “pain + male mouse” group (right) in 0~5 min and 30~35 min in the open field. **c** Neuronal staining profiles in the ACC of mice in the two groups. *Arc* (green) and *Homer1a* (red) foci were detected in nuclei (DAPI-labeled, blue). Scale bar, 30 μm. **d** Sample pie figures of the percentages of double-labeling, *Arc*/*Homer1a* single-labeling neurons in DAPI^+^ neurons in ACC of the two groups. **e** Statistic result of the percentages of double-labeling, total *Arc*/*Homer1a*-labeling neurons in DAPI^+^ neurons in ACC of the two groups. *n* = 5 for “pain + female mouse” group; *n* = 3 for “pain + male mouse” group. *p < 0.05, **p < 0.01, n.s. not significant

We used an animal model of persistent inflammatory pain to induce pain [13]. We injected 5% formalin for 5 μl into the left hind paws of adult male mice which were well habituated before the experiment (Fig. 1a). After the injection, mice were put into the open field, where there is a small cage in the center. Significant biting or licking behaviors were observed during the first 5 min, after that, mice moved less in the open field and preferred to stay in the corners. (Fig. 1b). At 30 min later, a female or male mouse was introduced and put into the central cage (Fig. 1b). The behavior of the male mouse was monitored for another 5 min. We found that male mice spent most of their time surrounding the female mouse, and tried to enter the central cage (Fig. 1b left). By contrast, they showed less interest to the male mouse (Fig. 1b right). Mice were sacrificed immediately at 35 min, and the brains were removed rapidly and quick-frozen. 20 μm-thick sections were prepared and mounted on slides. The catFISH process was carried out according to previous protocols [10, 11, 14]. In brief, riboprobes were transcripted from the *Arc/Homer1a* cDNA clone and labeled by digoxigenin and fluorescein individually. After hybriding with *Arc/Homer1a* mRNA on the slices in situ, anti-fluorescein/digoxigenin HRP and tyramide/cyanine-3 substrate of HRP were used to detect the *Arc/Homer1a* probes. Nuclei were counterstained with DAPI. While double-labeled neurons were detected in both the “pain + female mouse” and the “pain + male mouse” groups, we found that the percentage of ACC double-labeled neurons was larger in the “pain + female mouse” group than that of “pain +male mouse” group (Fig. 1d, ***p < 0.001, chi-square test). The percentage of *Arc*^+^ neurons, which indicates the sexual attraction activated neurons, was also higher in the “pain + female mouse” group than the “pain + male mouse” group (Fig. 1c, d, e, *n* = 5,3 respectively; **p < 0.01). The percentage of *Homer1a*^*+*^in ACC neurons show no significant difference (Fig. 1c, d, e, *n* = 5,3 respectively, *p* > 0.05).

In conclusion, by applying the *Arc*/*Homer1a* catFISH, we discovered that neurons encoding pain and sexual attraction information can be co-labeled in the same neurons of the ACC, which may indicate that pain and sexual attraction may be processed by some of the same neurons in the ACC. The molecular and synaptic mechanism still require further investigation.

## Abbreviations

ACC: Anterior cingulate cortex
catFISH: Cellular compartment analysis of temporal activity by fluorescent in situ hybridization
DAPI: 4,6-diamidino-2-phenylindole
IEG: Immediate early gene
